# Fuel for Fun: a cluster-randomized controlled study of cooking skills, eating behaviors, and physical activity of 4th graders and their families

**DOI:** 10.1186/s12889-016-3118-6

**Published:** 2016-05-26

**Authors:** Leslie Cunningham-Sabo, Barbara Lohse, Stephanie Smith, Ray Browning, Erin Strutz, Claudio Nigg, Meena Balgopal, Kathleen Kelly, Elizabeth Ruder

**Affiliations:** Department of Food Science and Human Nutrition, Colorado State University, 234 Gifford Building, Fort Collins, Colorado 80523-1571 USA; Wegmans School of Nutrition and Health, Rochester Institute of Technology, Rochester, New York 14623 USA; Department of Food Science and Human Nutrition, Colorado State University, 1571 Campus Delivery, Fort Collins, Colorado 80523-1571 USA; Nike, Inc., Beaverton, Oregon 97005 USA; Aims Community College, 5401 W 20th, Greeley, Colorado 80634 USA; Department of Public Health Studies, University of Hawaii at Manoa, 1960 East-west Road, Honolulu, Hawaii 96822 USA; Department of Biology, Colorado State University, 1878 Campus Delivery, Fort Collins, Colorado 80523-1878 USA; Department of Marketing, Colorado State University, 1278 Campus Delivery, Fort Collins, Colorado 80523-1278 USA

**Keywords:** Children, Cooking experience, Physical activity, Fruits and vegetables, Elementary schools, Parents, Experiential education, Eating competence, Online nutrition education, Family nutrition education

## Abstract

**Background:**

Childhood obesity remains a serious concern in the United States and in many other countries. Direct experience preparing and tasting healthful foods and increasing activity during the school day are promising prevention approaches. Engaging parents and families remains an important challenge. *Fuel for Fun: Cooking with Kids Plus Parents and Play* is a multi-component school- and family-based intervention for 4th graders and their families intended to promote positive food and activity environments, policies and behaviors at the individual, family and school levels. This paper describes the design and evaluation plan.

**Methods/Design:**

Four cohorts of 4th-graders and their parents from 8 schools in 2 districts in the same Northern Colorado region are participating in a 4-arm cluster randomized controlled trial. Theory-based *Fuel for Fun* consists of 5 components delivered over 1 school year: 1) *Cooking with Kids - Colorado*; an experiential classroom-based cooking and tasting curriculum, 2) *Cafeteria Connections*; cafeteria-based reinforcements of classroom food experiences using behavioral economic strategies, 3) *SPARK* active recess; a playground intervention to engage children in moderate to vigorous activity, 4) *Fuel for Fun Family*; multi-element supports targeting parents to reinforce the 3 school-based components at home, and 5) *About Eating*; an online interactive program for parents addressing constructs of eating competence and food resource management. Outcomes include child and parent measures of fruit and vegetable preferences and intake, cooking, physical activity, sedentary behaviors and attitudes. School level data assess lunch plate waste and physical activity at recess. In-depth diet and accelerometry assessments are collected with a subsample of parent-child dyads. Data are collected at baseline, immediately post-intervention at 7 months, and at 12 month follow-up. We anticipate recruiting 1320–1584 children and their parents over the length of the project.

**Discussion:**

The *Fuel for Fun* study design allows for impact assessment of school-, family- and online parent-based intervention components separately and in combination. Study strengths include use of theory- and evidence-based programs, valid child and parent self-report instruments, and objective measures of food, cooking, and physical activity behaviors at the individual, family and school levels. Parent involvement and engagement is examined through multiple strategies.

**Trial registration:**

Clinicaltrials.gov registration number NCT02491294. Registered 7 July, 2015.

## Background

Childhood obesity continues to be a major public health concern in the U.S. and elsewhere. School-based interventions addressing diet and physical activity have demonstrated promise [1]. Specifically, programs that include children in cooking and food preparation and increase physical activity during the school day are recommended to counter childhood obesity [2–4].

A recent systematic review of child-centered cooking programs identified eight articles meeting the inclusion criteria [5], of which two were rated strong in quality [6, 7]. Both of these highly-rated studies were evaluations of the *Cooking with Kids* (*CWK*) curriculum, a school-based food and nutrition education curriculum that engages elementary school children in hands-on learning with fresh affordable foods based on diverse cultural traditions (http://cookingwithkids.org/) [8]. Implementation of the full curriculum includes 16 h of cooking and tasting lessons throughout the school year, but allows for modifications to accommodate fiscal, time, and resource limitations. Participation in *CWK* among predominantly Hispanic, 4^th^ grade children demonstrated gains in cooking self-efficacy among children with no previous cooking experience, and demonstrated increased preference for fruits and vegetables, especially for students receiving the cooking and tasting curriculum (as opposed to the curriculum with only tasting lessons). Moreover, gains in self-efficacy were particularly pronounced in male students [7]. Self-efficacy in cooking and fruit and vegetable preferences are relevant measures given that self-efficacy related to diet and nutrition is strongly correlated with dietary behavior [9], and fruit and vegetable preferences are often associated with intake [10, 11]. Similar gains with *CWK* engagement were documented in a predominately non-Hispanic white sample of 4^th^ graders [6]. In that investigation of a *CWK* program of shorter duration (10 h of cooking and tasting lessons), significant improvements in vegetable preferences, and cooking and food preparation attitudes and self-efficacy were affirmed with *CWK* participation. As with the previous *CWK* investigation, students who reported no prior cooking experience demonstrated the greatest improvement in these measures, and the effect was greatest in boys. Together, these studies support participation in *CWK*, and affirm the positive influence of a curriculum designed to allow students of diverse cultural backgrounds direct experience to foods through cooking or tasting activities.

Physical activity (PA) is the other cornerstone to energy balance. The school environment plays an important role in children’s PA accumulation, but despite opportunities for PA during recess breaks and physical education classes, the sedentary nature of many school environments poses a challenge to school-day PA accumulation [12]. Interventions and other studies have reported that increasing school day PA accumulation increases whole-day PA in a magnitude greater than expected from the changes in school-day PA alone [4]. For instance, children encouraged to be physically active during the school day (e.g., during recess and physical education) have been shown to be more active after school than children whose PA was restricted during school [13]. *Sports Play and Active Recreation for Kids* (*SPARK*) is one program shown to increase students’ moderate to vigorous PA during the school day [14].

Although energy balance may be as simple as “calories in” versus “calories out” from a physiologic standpoint, translating this principle to achieve improvements in public health is more complex. Successful programs and interventions aimed at obesity prevention must also address the context in which an individual’s nutrition and physical activity choices are made [15, 16]. For a pediatric audience, this includes the individual child, parents and family, school (including cafeteria) and community [17, 18]. This type of system-wide approach engages multiple sectors of society to improve health and reduce overweight and obesity [15]. This approach stems from the Social Ecological Model (SEM) [19], and is central to the action plan for the Dietary Guidelines for Americans 2015–2020 [20].

Fuel for Fun: Cooking with Kids Plus Parents and Play (*FFF*) is one such multiple-component, school and family intervention with a social-ecological approach. *FFF* incorporates the tested, validated *CWK* program into wider school environments to include the cafeteria and facilitate family involvement. *FFF* builds on prior research to engage multiple sectors of the community to reduce the risk of childhood obesity by promoting healthful food and activity environments, policies and behaviors through: 1) developing and testing the efficacy of a 4th grade comprehensive school- and family-based intervention, 2) applying this intervention to an after-school setting, and, 3) state-wide program dissemination. Two of these aims, applying this intervention to an after-school setting, and to a state-wide program dissemination, will not be described here. The purpose of this paper is to describe the study protocol for the *FFF* school- and family-based intervention.

## Methods/Design

**Fig. 1:**
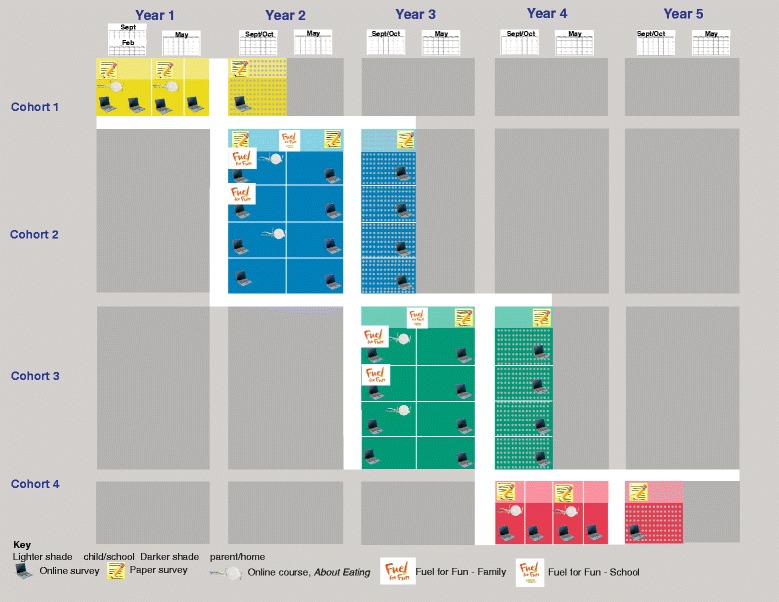
*Fuel for Fun*: Cooking with Kids Plus Parents and Play Research Design. Fourth-grade students and their parents participate in this research study

*Fuel for Fun* builds on our prior research and tenets of the Satter Eating Competence Model, Social Cognitive Theory (SCT), Experiential Learning Theory (ELT), SEM, Behavioral Economics, and Social Marketing [19, 21–25]. Over 4 years, annual cohorts participate in a 4-arm cluster randomized controlled trial that includes post-intervention follow-up. As shown in Fig. 1, cohorts 1 and 4 represent non-treatment with treatment occurring in cohorts 2 and 3.

Eight schools from 2 districts in the same Northern Colorado county were matched on percent of students receiving free/reduced price school meals, then randomly assigned to 1 of 4 arms so that 2 schools are in each of the following arms:

1. classroom, cafeteria, and playground components;
2. classroom, cafeteria, and playground components with multi-element family component;
3. classroom, cafeteria, and playground components with online parent education; and
4. classroom, cafeteria, and playground components with multi-element family component and online parent education.

### Setting and participants

**Fig. 2:**
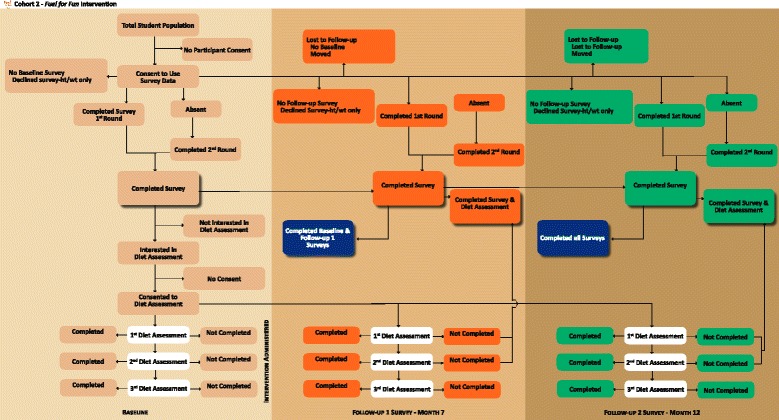
Fuel for Fun child participation flowchart

**Fig. 3:**
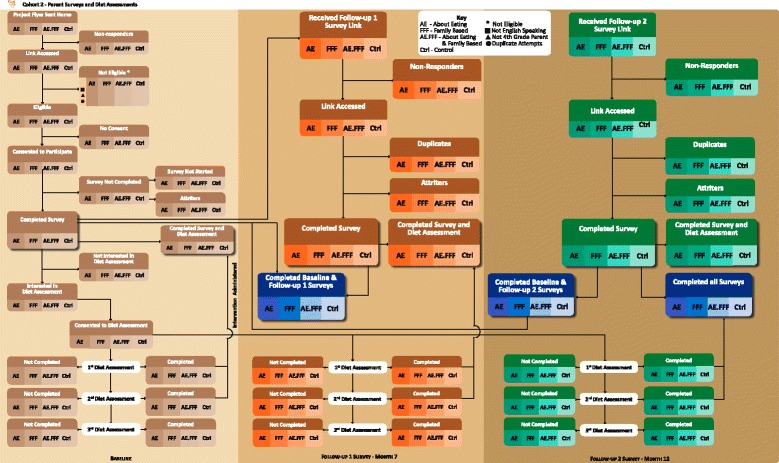
Fuel for Fun parent participation flowchart

Approximately 55 4^th^ grade students are enrolled in each school annually. We anticipate 75–90 % participation, thus totaling 330–396 students for each of the 4 years (1320–1584 over the length of the project). In one school district, 74 % of students are white, 18 % Hispanic, 3 % Asian, and 1 % African-American. In the second school district 75 % of students are white, 20 % Hispanic, 1 % Asian, and 1 % African American. Three of these 8 schools are assigned to participate in accelerometry assessment, and 100 parent-child dyads are recruited from all participating schools for in-depth diet assessment. Consort charts for survey and diet assessment measures are shown in Figs. 2 and 3 for child and parent participation, respectively.

### Ethical approval and consent

The study is approved by the Colorado State University, the Pennsylvania State University, and the Rochester Institute of Technology Institutional Review Boards and entities with similar functions within both school districts. All participating students and their parents sign assent/consent forms.

### Intervention

**Fig. 4:**
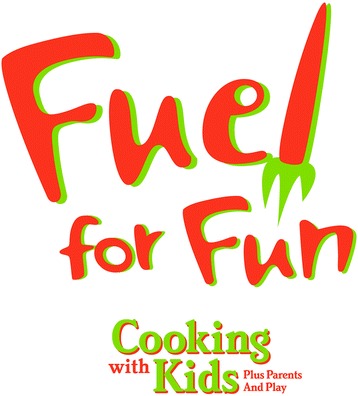
*Fuel for Fun*: Cooking with Kids plus Parents and Play Logo

Co-investigators with branding expertise applied marketing strategies to generate a unique project name and logo. Name and logo selections were tested for understanding, clarity, and likeability by parents and children similar to the target audience. In testing, youth and adults described the logo as representing the scope of the project (i.e., “shows action and nutrition” in attention-demanding colors). After confirming no copyright infringement, we adopted *Fuel for Fun: Cooking with Kids plus Parents and Play* (Fig. 4) as the project name and logo.

**Fig. 5:**
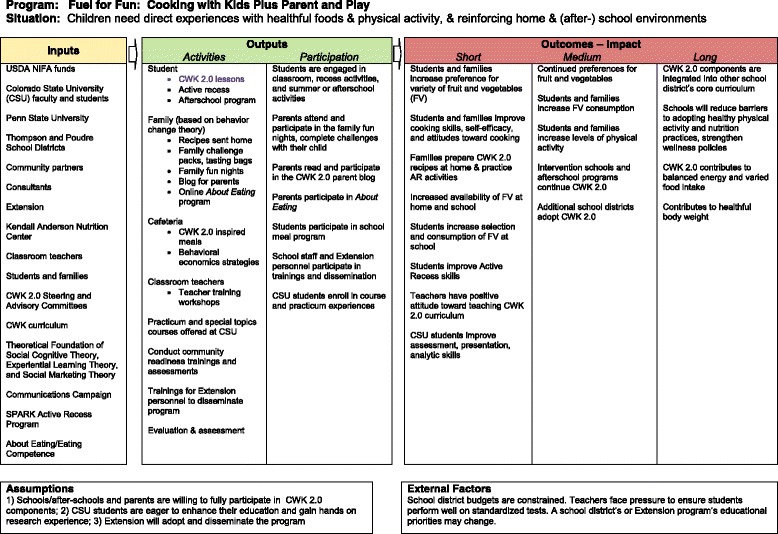
*Fuel for Fun*: Cooking with kids plus parents and play logic model

*Fuel for Fun* consists of 5 components: 1) *Cooking with Kids - Colorado*; an experiential classroom-based cooking and tasting curriculum, 2) *Cafeteria Connections*; cafeteria-based reinforcements of classroom food experiences using behavioral economic strategies, 3) *SPARK* active recess; a playground intervention to engage all children in moderate to vigorous activity [26], 4) *Fuel for Fun Family*; multi-element supports to reinforce the 3 school-based components at home targeting parents, and 5) *About Eating*; an online interactive program for parents addressing constructs of eating competence and food resource management [21]. Development and mode of delivery of each component is described below. The project logic model is depicted in Fig. 5.

### Intervention development

The development of *Fuel for Fun* uses 3 strategies: 1) Design and development of new components; 2) adaptation of existing, tested activities and curricula; 3) integration of existing, stand-alone programs.

The study is adapted from and expands upon prior research examining the efficacy of the Cooking with Kids, Inc. program (*CWK)* [6, 7, 27]*.* For the experiential nutrition classroom component, the original *CWK* cooking and tasting lessons were reviewed and revised to more closely align with the state of Colorado academic standards and nutrition and education best practices. This 8-month process involved a K-12 interdisciplinary curriculum expert, 5 educators and curriculum specialists from the participating school districts, the *CWK* curriculum developers, and project nutrition education experts. Substantive changes included revising the lesson structure to be consistent with the Understanding by Design format [28] so that assessment strategies consistently address learning objectives and standards; greater emphases on in-depth nutrition and mathematics knowledge and skills; regionally-relevant content (e.g., letters from local farmers); and more opportunities for differentiated instruction for diverse learning needs [29].

Integrating experiential classroom nutrition activities with cafeteria meals can increase fruit and vegetable consumption [30–32]. The *FFF* cafeteria component – *Cafeteria Connections* – uses behavioral economic strategies [24, 33] to connect what children learn in the *FFF* classroom cooking and tasting lessons to foods served as part of the National School Lunch Program.

A companion family component was developed to reinforce the school-based intervention. It incorporated successful strategies identified through an extensive literature review, and included: family nights at school, home-based action packs (i.e., activity packets) for parent and child to complete together, and a parent blog. Subsequent telephone interviews with a representative sample of parents (*n* = 24; 41.7 ± 5.7y, 92 % female, 91 % White, 38 % used ≥ 1 food assistance program) of non-intervention 4^th^ graders explored these elements in greater detail. Parents preferred family events at school to be held toward the end of the work week (Thursdays or Fridays) and in the early evening. Topics of greatest interest included ways to be more physically active as a family, easy-to-fix meals, strategies to prepare food with their child/ren, and affordable home meals [34].

From these interview results we developed a family night protocol and procedures manual adapted from other school-based studies [35, 36]. Cooking stations and physical activities were designed using several constructs from SCT and ELT [22, 23]. We held 2 pilot events at a non-participating school, with half of the events tested at the first event and the remainder at the second. Surveyed parents and children gave the event high marks (e.g., ≥ 4.0 on 5 point scales, data not shown), and process evaluation through observation and debriefing after each event identified minor revisions (e.g., refining necessary booth resources and improving event flow). Action packs and parent blogs were designed with educator and social media expert input respectively, to reinforce the content of the classroom cooking and tasting lessons and *SPARK* recess games. Final content of each intervention component is described below.

#### Classroom

The *FFF* classroom component - *Cooking with Kids Colorado* - includes 5 2-h *CWK* cooking and 5 1-h tasting lessons taught over the course of the academic year (i.e., 1 lesson approximately every 3 weeks). Lessons emphasize experiential learning of practical cooking skills using fresh and affordable foods from diverse cultural traditions [27]. Participating teachers are invited to an orientation session prior to the commencement of the program. Cooking and tasting lessons are taught in the classroom or cafeteria by graduate nutrition students trained as Food Educators, with assistance from the classroom teacher and another (graduate or undergraduate) nutrition student.

#### Cafeteria

*FFF Cafeteria Connections* includes lunch service of fruit and vegetable items that children prepare and taste in the classroom lessons, placing these fruits and vegetables prominently on the lunch line, highlighting them with colorful Fuel for Fun branded signage, and use of verbal prompts to encourage students to select fruits and vegetables. Cafeteria staff are trained to deliver these intervention components during the weeks that cooking and tasting lessons are delivered in the 4^th^-grade classrooms, and they also wear FFF chef coats or aprons on school days when implementing these strategies to further promote the program.

#### SPARK active recess

Since all 4^th^-grade students in the eight schools participate in lunch recess for approximately 20 min each day, this recess period offers an ideal opportunity for children to increase their physical activity without taking away from the school curriculum. Sports, Physical Activity and Recreation for Kids (*SPARK*) is an evidence-based program shown to increase children’s participation, confidence and skills in physical activity [26]. Trained Active Recess Leaders (university Health and Exercise Science students) lead *SPARK* Active Recess activities a minimum of 4-days a week on the school playground during lunch recess. The noncompetitive games include “sharks and minnows”, “2 square” and “cat and mice.” Schools are provided with all activity equipment that supports the Active Recess program, such as balls, hoops, cones, jump ropes, bean bags, and flying discs.

#### Fuel for Fun Family

*FFF Family* is offered in those schools receiving the family component and includes three elements: Family Nights, Action Packs, and a parent blog. The *FFF* Family Night is scheduled two times each intervention year, fall and spring, and takes place at the participating school. Invitation flyers are sent home with students at least two weeks in advance, and two additional email reminders are sent to parents before the event. Activities include cooking and tasting stations that mimic what students experience in the classroom, food and nutrition crafts, and *SPARK* games children have learned at recess. After families rotate through each station, they are invited to enjoy a light meal provided and served by the school cafeteria staff. Meals are designed using project recipes. To motivate attendance and promote project goals, each Family Night closes with a drawing for free cooking and activity prizes.

The second family element, *FFF* Action Packs, is an activity packet sent home with students after each tasting and cooking lesson. The activities correspond with each classroom lesson and *SPARK* Active Recess games and include nutrition and physical activity sections. Participation of parents and other family members is strongly encouraged through written prompts and documented by parent signature. Students are reminded to return completed Action Packs by the following lesson. Lastly, the *FFF* Parent Blog is designed to engage parents and keep them up-to-date on the *FFF* activities going on in their child’s school. The blog, which is slightly tailored for each school to align with the dates of *FFF* activities, also provides parents with tips about cooking with children, encouraging children to try new foods, and physical activities for the family. Content is posted to the private Facebook page weekly during the intervention, with reminders sent to parents to view the posts.

#### About Eating

*About Eating* is a web-based, self-directed, interactive program that addresses core constructs of the Satter model of eating competence [37], and focuses on food enjoyment and acceptance, attention to internal regulation, food resource management skills, and physical activity. It is learner-centered in that each of the 6 lessons can be viewed in any order, as often as desired, and with individually tailored depth and scope of participation. Iterative development and evaluation activities reveal that *About Eating* has face, process and content validity [21]. *FFF* parents in schools assigned to receive *About Eating* are invited to begin this program after they have completed their first survey (https://www.needscenter.org/resources1/about-eating/).

### Assessment and outcome measures

A description of each measure is provided below. Refer to Table 1 for a list of measures and timing of data collection at the individual (child, parent), classroom, cafeteria, and playground levels.

**Table 1:** Evaluation measures for children and parents participating in the *Fuel for Fun* study^a^

Target measurement	Instrument/Process	Description	Child	Parent
	Individual Level			
Demographics	Child age, birthdate, gender, race and ethnicity; parent gender, age, race and ethnicity, nutrition and food assistance program participation, level of schooling, serious disease diagnosis	Child information obtained from class rosters provided by schools; parent information is self-reported as part of an online parent survey	X	X
Height/weight	Child measured; parent self-report	Child data collected by research team using standard protocol; parent self-reported as part of online survey	X	X
Dietary intake assessment (24-h recalls)^b^	Student-telephone	The Pennsylvania State University Diet Assessment Center protocol	X	X
	Parent-online			
Physical activity	7-day accelerometry (75 hz; GENEActiv)^c^	7 days of free living, wrist-mounted accelerometry data from children and their parents; customized Matlab program will sum child and parent accelerations over 1 and 60 s, respectively, and apply published GENEActiv cutpoints to determine amount of time in MVPA weekday, weekend day, and specific time periods (before school, school-day, after-school, and evening)	X	X
	Minutes/week of moderate-to-vigorous physical activity (MVPA); adaptation of Godin/Shephard questionnaire [62]	Students asked days/week and minutes/day of vigorous, moderate, and mild activity during free time; responses for vigorous and moderate PA summed for total MVPA.	X	
	Screen time	Numbers of hours spent/day watching TV, playing video games or using a computer (not for homework). Responses 0–11 h	X	
	Stage of change for regular physical activity [42, 43]	Students asked “Do you do regular physical activity as described?” Each of 5 responses correspond to one of the stages of Transtheoretical Model	X	
	International Physical Activity Questionnaire (IPAQ) [63]	Responses converted to met-min/week and identified as low, moderate, and high activity categories		X
Cooking experience	Cooking with Kids Student Survey [38]	Do you cook with family? Do you cook with friends? Do you cook? yes or no response options	X	
Fruit and vegetable preferences	Cooking with Kids Student Survey	Preference for 7 fruits and 11 vegetables; 18 items, 5 response options, scored from 1 to 5, possible score 18–90. Cronbach’s α 0.82	X	
Cooking self-efficacy	Cooking with Kids Student Survey	Self-efficacy for skills related to cooking; 8 items, 5 response options, scored from 1 to 5, possible score 8–40. Cronbach’s α 0.70	X	
Cooking attitudes	Cooking with Kids Student Survey	Attitude toward cooking and making food; 6 items, 5 response options, scored from 1 to 5, possible score 6–30.Cronbach’s α 0.76	X	
Eating Competence:	Satter Eating Competence Inventory (ecSI 2.0) [44]	Parents: 16 items, 5 response options scored from 3 to 0. Possible score 0–48; scores 32 indicate eating competence. Cronbach’s α 0.89	X	X
		Students: FU1 3 Eating attitudes and behavior items; possible score 0–9		
		FU2 16 items, 5 response options scored from 3 to 0 Possible score 0–48; scores 32 indicate eating competence. Cronbach’s α 0.89		
Food resource management skills	Expanded Food and Nutrition Education Program (EFNEP) adults core behavior checklist questions [64]	13 items, 5 response options. Mean value for each item		X
Culinographics (cooking practices demographics)	Questions from Krall and Lohse [44]	7 items, multiple choice		X
Modeling eating behavior	Modeling Scale. Sample items: How often do you eat dinner with your child?	11-items from modified scale, each with 4 response options. Possible scores 0–33. Cronbach’s α 0.77		X
	How often do you eat vegetables at dinner with your child? [65]			
Self-efficacy/Outcome expectancies (SE/OE)	Perceived ability to offer fruits and vegetables that their child will eat. Sample item: I can prepare vegetables that my child will like. [66]	12-items modified from tested measure each with 5 response options. Possible scores 12–60. Cronbach’s a 0.93		X
In-home fruit and vegetable availability	Fruit and vegetable availability inventory. [46]	20 items (fresh, frozen, canned fruits, vegetables and 100 % juices) listed. Availability was affirmed or denied. Possible scores 0–20		X
Parenting Style	Caregiver’s Feeding Style Questionnaire [67]	19 items, 5 response options. Scores converted to 4 caregiver feeding styles.		X
Parent Perceived Stress	Single item from the Community Health Database [68]	Visual analog scale from 0 (no stress) to 10 (extreme stress).		X
	Group Level			
Plate waste assessment^d^	Digital photography [51]	Pre-consumption reference trays and post consumption trays photographed; plate waste of each food item estimated to nearest 10 %	X	
Physical activity assessment/observation^e^	SOPLAY observation [26]	Validated tool for direct observation of physical activity associated and environmental characteristics in free play settings. MVPA and estimates of energy expenditure are calculated from activity counts	X	

^a^Measures collected at Baseline, month 7 and month 12

^b^Dietary intake assessment completed with a subsample of up to 100 parent/child dyads

^c^Accelerometry measured on a subsample of children and parents from 3 of the 8 participating schools

^d^Plate waste estimated from lunches of all assenting 4th-grade students participating the National School Lunch Program

^e^System of Observing Play and Leisure Activity in Youth; conducted at all 8 participating schools during lunch time recess

#### Child measures

##### FFF student survey

The *FFF* student survey in each participating classroom at 3 time points: Baseline (at the beginning of the school year prior to intervention, e.g., September), Follow-Up 1 (after intervention is completed in late spring of the same school year, e.g., May), and Follow-Up 2 (fall of the following school year, e.g., October). Standardized administration includes instructions and items read aloud as students complete the survey using pencil and paper. This survey set includes 35 cooking and fruit/vegetable items previously confirmed for validity and reliability [38]. Seven of these items address students’ fruit preferences, 11 items for vegetable preference, 6 items for cooking attitudes, 8 items for cooking self-efficacy, and 3 items assess prior cooking experience.

Student report of physical activity is assessed through a 6-item adaptation of Godin & Shephard’s brief Leisure-Time Exercise Questionnaire [39, 40], measuring days per week and daily minutes of mild, moderate and strenuous activity. Sedentary behavior is measured through students’ estimate of daily hours of TV watching, video game playing and use of the Internet for non-homework activities [41]. PA stage of change is determined using Schumann and colleagues’ measure [42, 43].

##### Height and weight

Aligned with the *FFF* survey administration period, all participating children’s height and weight are measured by trained research staff. Height is measured to the nearest tenth of a centimeter using a portable stadiometer (SECA, Model 213), and weight measured to the nearest tenth of a kilogram using a standard scale (Health o meter, Model 394KLX). Children are instructed to remove their shoes and any heavy clothing (e.g., jackets, sweatshirts tied around their waists, etc.) prior to measurement. Height is measured with children’s arms by their sides and looking straight ahead. The research team returns within one week to collect measures from students absent on the day of testing.

#### Parent measures

##### FFF parent survey

Consented parents complete an online survey at the same 3 time points as their child. For the first and second follow-up surveys, email reminders are sent within 2 to 4 weeks after the initial “evite.” The survey includes demographics and valid measures of eating competence, [44], sense of coherence [45], modeling eating behaviors, self-efficacy, and fruit and vegetable availability [46]. Upon survey submission, parents are sent an animated, musical e-card with an e-gift card pin.

#### Physical activity assessment of parent-child pairs using accelerometry

Fourth-grade children and their parents in 3 randomly selected *FFF* schools are recruited to participate in the accelerometry (ACC) portion of the study. Using the wrist-mounted GENEActiv ACC recording at a sampling frequency of 75Hz (ActivInsights Ltd., Cambridgeshire, UK), seven days of free-living data are collected. The device is attached to each child’s non-dominant wrist using a semi-removable, hospital-style plastic band. Children are instructed to leave the device on for the entire seven-day period, even while sleeping and bathing. Parents and teachers are provided written instructions for children’s ACC use, notably, that children should not tamper with the device nor remove it during the assessment period. Children are encouraged to engage in their typical daily activities while wearing the ACC, and teachers provide the research team a daily class schedule for the week of ACC wear, including school start and end times, lunch and recess times, and PE periods. Extra wristbands are provided in case a child has to remove the ACC for any reason. Children receive a $10 gift-card to a local “superstore” upon receipt of the ACC as compensation for participation. Accelerometers, written instructions, and gift-cards for any consented children absent at drop-off are left with the teacher, with instructions to attach the device upon the child’s return to school. At the end of the seven-day period, members of the research team return to collect the ACCs and the children receive a second gift card, this one valued at $15. Children must return their device and the device worn by their parent, if applicable, to receive the $15 gift card.

Parents who consent for themselves and their child to the ACC portion of the study receive a packet with one ACC, semi-removable plastic ACC wristbands, and ACC instructions, which detail how to attach the device and frequently asked questions regarding the ACC and ACC-wear. This packet is sent home with the child on the ACC drop-off day, and children are asked to give the ACC to their parents as soon as they get home. Similar to the child protocol, parents are asked to wear the device for seven days and to not tamper with or remove the ACC. Parents are instructed to return the device to school via their child on ACC pick-up day. There is no separate compensation for parents to participate in the ACC portion of the study. These parents are also asked to complete a demographic information form surveying their sex, height, weight, and age.

#### Diet assessment of parent-child pairs using 24-h dietary recalls

At the baseline of project years 2 through 4, following completion of the parent survey, we are recruiting parents to participate with their child in a diet assessment (DA) protocol for each of the three data collection time points described above. Child DA consists of three 24-h dietary recalls collected by telephone over a 2–4 week period by trained interviewers at the Pennsylvania State University Diet Assessment Center. Intake data are collected using Nutrition Data System for Research software versions 2013 and 2014, developed by the Nutrition Coordinating Center (NCC), University of Minnesota, Minneapolis, MN (http://www.ncc.umn.edu). This time-related database updates analytic data while maintaining nutrient profiles true to the version used for data collection [47]. These child-oriented interviews are conducted with the parent present in most cases, and typically last 15 to 20 min. Calls are unannounced with a goal of obtaining two weekday and one weekend dietary recalls.

Upon completion of three telephone dietary recalls for the child’s intake, we contact parents via email to complete online dietary recalls for themselves using the National Cancer Institute's Automated Self-administered 24-h recall (ASA24) software [48–50]. Email requests are also unannounced with a goal of obtaining two weekday and one weekend dietary recalls. If parents are unresponsive to email requests, telephone contact is made to ensure they were receiving the requests and to encourage participation. Parent dietary intake data are collected and analyzed using the ASA24 system, version 2011 [50]. We send parents e-gift cards for each DA completed; values increase incrementally for each of the 3 DA. E-gift card pins are embedded in an online animated, musical card.

#### School-level outcome measures

##### Plate waste assessment

To assess the impact of *FFF* on student fruit and vegetable intake during school lunch, the selection and consumption of all foods obtained from the school cafeteria during the 4^th^-graders’ lunch period is measured using a tested digital photography plate waste method [51–54]. Plate waste is assessed on four occasions throughout the school year; once at baseline and three times after implementation of the intervention. Only fourth-graders purchasing a National School Lunch Program reimbursable meal (approximately 50 % of all 4^th^-graders) are invited to participate. Photographs taken of five samples of each menu item serve as the “before” reference photographs. Two independent analysts estimate the difference of each menu item from the “after” photo of each student’s lunch tray compared to the “before” photos to the nearest 10 %.

##### System for Observing Play and Leisure Activity in Youth (SOPLAY)

We use the SOPLAY procedure [55] to document the number of students and their physical activity levels on the school playground during lunch recess. Each school playground is divided into distinct scan areas (e.g., swing sets, basketball court, open field). During a scan, each child’s activity is mechanically coded as sedentary (e.g., standing, sitting), walking, or very active (e.g., running, jumping). Separate scans are made for boys and girls. Predominant activity type (e.g., climbing, soccer) and characteristics of each area are noted, including accessibility, usability, and presence of supervision, organized activities and equipment. Time of day, day of week, temperature, weather conditions, and other variables that can impact the length of recess or children’s physical activity levels are also recorded. During each of three measurement periods for each cohort, four lunch time recesses are observed at each school over 10 school days (2 weeks), resulting in a total of 32 observation days per measurement period. Reliability data will be collected during eight of these 32 observation days (25 %) by pairs of observers who will make simultaneous and independent observations.

##### Teacher measures

After the completion of the intervention, teachers are emailed an invitation to complete an online survey. Survey items were adapted from a previously validated instrument for classroom teachers [56], with content validity confirmed by expert panel and face validity and comprehension confirmed through cognitive interviews with 4 elementary school teachers from the same district, but not participating in Fuel for Fun [57]. Survey items include beliefs about the school nutrition environment, including who is responsible for that environment, teachers’ abilities to influence the nutrition environment, and the importance of role modeling healthful nutrition behaviors. Other survey content includes eating competence [44], education level and years of teaching experience, cooking attitudes and behaviors and college nutrition course participation. Upon survey completion they are given a thank you note and $25 gift card.

#### Process measures

- Food Educators complete a debriefing form at the conclusion of each classroom cooking and tasting lesson to record numbers of students present, student recollections from the previous lesson, student and classroom teacher engagement, and any issues encountered or changes to the lesson.
- Research team members observe each Food Educator four times during the school year to assess fidelity to the lesson plan, any issues observed, and Food Educator, student and teacher engagement [58]. Clarification of expectations is provided to the Food Educator as needed to confirm consistency of lesson delivery.
- *SPARK* leaders complete a debriefing form after each active recess to document number of students, length of recess, weather conditions, *SPARK* activities implemented, and any issues encountered. Each *SPARK* leader is observed five times throughout the school year by a member of the research team to document fidelity to the activity plan.
- School cafeteria staff are observed by members of the evaluation team during lunch four times throughout the school year to document implementation of the Cafeteria Connection component.
- Sign-in sheets for students and their families document participation in Family Night events. The Family Night event coordinator records recruitment, fidelity to the planned activities, and overall impressions of the event. Parents and students complete a short survey addressing their attitudes toward the Family Night activities.
- Parent engagement in the Action Pack activities is assessed with a parent signature on each returned action pack and researcher review to determine number of action pack completions.
- Research staff monitor timing, content and participation in the parent blog posts.
- *About Eating* participation is monitored by website analytics and survey administered immediately after each lesson.
- District and school-level data are requested annually from wellness coordinators that describe the nutrition and activity environments at each school. These include length of lunch periods, frequency and length of physical education classes, whether recess occurs before or after lunch, and description of any nutrition education topics or programs taught during or after school.

### Analysis plan

Interview data and other qualitative process data are analyzed using Atlas.ti (Cleverbridge, Inc; Chicago, IL) and NVivo (QSR International; Burlington, MA) to conduct content and thematic analyses following transcription of audio records. SOPLAY data are ranked and categorized as suggested by McKenzie and colleagues [26]. Scores are calculated as directed for each adult and child survey. Following determination of or transformation to a normal distribution and assessment of survey internal consistency, data are analyzed using a repeated measures general linear model that controls for significant group (co-factor) or characteristic (covariate) differences. Cluster analyses will also be conducted to assist with data interpretation.

Power calculations for primary outcomes are based on previous studies with 4^th^ grade Colorado students and parents. Minimum sample sizes to detect a clinically relevant change at a power of 0.80 with an alpha of 0.05 are shown in Table 2 below. According to experts at The Pennsylvania State University Diet Assessment Center, datasets are useful when at least 75 individuals have completed at least 2 of the 3 diet recalls for each measurement period.

**Table 2:** Sample size requirements for power levels of 0.8 and 0.9 based on prior studies of *Cooking with Kids*

Measure	Clinically relevant change	Minimum sample size/group^a^	
		1 - *β* = 0.8	1 - *β* = 0.9
Student attitude	2	50	66
Student self-efficacy	2	108	144
Student FV preference	3	127	170
Parent modeling	2	64	86
Parent self-efficacy	4	110	146
Parent FV availability	2	15	20
Parent eating competence	3	105	140

^a^Based on repeated pre/post measures using standard deviations from previous research. For example, a total of 100 participants are needed in this two-treatment parallel-design study. The probability is 80 % that the study will detect a treatment difference in student attitude at a two-sided 0.05 significance level, if the true difference between treatments is 2 units. This is based on the assumption that the standard deviation of the response variable is 3.5

website: http://hedwig.mgh.harvard.edu/sample_size/js/js_parallel_quant.html

#### Accelerometry

Child ACC data are downloaded with the GENEActiv PC Software (ActivInsights Ltd., Cambridgeshire, UK). Using a custom R script (R Foundation for Statistical Computing, Vienna, Austria), data are low-pass filtered at 15-Hz, vector summed, and corrected for gravity by subtracting one. Values are then averaged over 75Hz to obtain one value per second of wear-time. Next, a custom Matlab program (Mathworks, Inc., Natick, MA) and previously determined cutpoints [59] are used to process the acceleration data and output minutes spent in activities of sedentary, light, moderate, and vigorous intensities for each day that the device was worn and for specific intervals of the day (i.e., full-day, before school, school-day, recess, PE, after-school, evening). This program also identifies periods of time when the device was not worn (any period of >60 min during which ACC output was <0.06 gs or any period that was identified as having ≥98 % of the interval indicated as sedentary). To be included in analyses, children are required to have at least four valid days of ACC-wear, with a valid day including at least 600 min of ACC wear-time. Any days with less than 600 min of wear and any children with fewer than four valid days are removed from the dataset.

Like the child data, parent ACC data are downloaded with the GENEActiv PC Software (ActivInsights Ltd., Cambridgeshire, UK), and a custom Matlab script (R Foundation for Statistical Computing, Vienna, Austria) is used to vector sum and correct the data for gravity by subtracting one. Values are then summed over 60-s to obtain one value per minute of wear-time. Next, a custom Matlab (Mathworks, Inc., Natick, MA) program and previously published cutpoints [60] are used to process the acceleration data and output minutes spent in activities of sedentary, light, moderate, and vigorous intensities for each day that the device was worn and for specific intervals of the day (i.e., full-day, before school, school-day, after-school, evening, late-evening). This program also identifies periods of time when the device was not worn (any period of >60 min during which ACC output was <100 gs or any period that was identified as having ≥98 % of the interval indicated as sedentary). To be included in analyses, parents are required to have at least four valid days of ACC-wear, with a valid day including at least 600 min of ACC wear-time. Any days with less than 600 min of wear and any parents with fewer than four valid days are removed from the dataset.

#### Quality control and data management

Research staff and university students are trained in intervention implementation and/or data collection and entry as appropriate. Student survey and height/weight data are dual entered, then compared using SPSS by a third research team member to confirm accuracy. Parent data are downloaded from Qualtrics [61], reviewed to assure eligibility and compared with incentive payment records. All datasets are maintained on a password protected, secure University server.

## Discussion

The *Fuel for Fun*: Cooking with Kids Plus Parents and Play logic model depicts the breadth of resources, inputs, and activities designed to achieve immediate and longer-term outcomes to effectively prevent childhood obesity within study participants (Fig. 5). The study design (Fig. 1) includes 4 annual cohorts (2 student control and 2 intervention) in 8 schools from 2 school districts, with 2 parent groups in the control cohorts and 4 parent groups in the intervention cohorts. All students receive the school-based classroom, cafeteria, and active recess components. Parent group involvement includes a range of activities from control to both *FFF* family involvement and *About Eating*, an online food resource management program.

Unique strengths of this study include significant opportunities for parent involvement, and examination of parent engagement and parent response to 1 of 4 treatment options. Other significant contributions include the use of accelerometry, dietary, and survey data; with parent-child dyads for accelerometry and dietary assessments. Unlike many childhood obesity prevention interventions, we will follow students and parents into the subsequent school year, collecting a full year of diet and physical activity-related information. Additionally, the intervention and control groups are randomly assigned and cohort-based. Our dissemination plan includes adapting and testing *FFF* in afterschool and other out-of-school based venues and for low-income audiences.

This project builds on our prior work that shows impact of the *CWK* intervention on 4th graders, especially boys without previous cooking experience for both Hispanic [7] and mainly Caucasian samples [6]. In addition, eating competence, shown to be associated with parent feeding and food-related behaviors in prior Hispanic sample [46], is included in the assessment of this mostly Caucasian, socioeconomically diverse sample of parents. *Cafeteria Connections* builds on previous school cafeteria behavioral economics research by Wansink and Just [24, 33] and ties the strategies to the *FFF* classroom lessons. Moreover, valid instruments and procedures are used to collect all study data.

In addition to examining the impact of an effective cooking and tasting curriculum on student food-related behaviors, we will examine parent engagement, interplay between parent and child involvement, relationships between physical activity, diet, and eating behaviors, and sustainability and dissemination potential of this type of curriculum. We will also explore and characterize the psychometric properties of previously tested instruments about physical activity, food and eating behaviors.

Limitations include that the target audience lacks racial and ethnic diversity, thereby restricting the generalizability of results. Because of the intervention design, we will have only indirect involvement of parents, relying on them to accept our invitations to join their child in intervention and measurement activities. If teachers continue teaching 4th grade in participating schools, they will participate in both control and intervention phases of the project; their prior experience may influence subsequent years. We will not be able to follow students beyond the beginning of the 5th grade year, therefore long-term impact is unknown.

## Abbreviations

ACC, accelerometry; ASA24, Automated Self-administered 24-h recall; *CWK*, Cooking with Kids; DA, diet assessment; ELT, Experiential Learning Theory; *FFF*, Fuel for Fun; PA, physical activity; SCT, Social Cognitive Theory; SEM, Social Ecological Model; SOPLAY, System for Observing Play and Leisure Activity in Youth; *SPARK*, Sports, Physical Activity and Recreation for Kids
